# Alveolar rhabdomyosarcoma of the anterior mediastinum with vessel invasion in a 4-month-old boy: a case report

**DOI:** 10.1186/s13256-015-0642-4

**Published:** 2015-07-17

**Authors:** Simon C.Y. Chow, Randolph H.L. Wong, Innes Y.P. Wan, Ka Fai To, Song Wan, Malcolm J. Underwood, Calvin S.H. Ng

**Affiliations:** 1 Division of Cardiothoracic Surgery, Department of Surgery, The Chinese University of Hong Kong, Prince of Wales Hospital, 30-32 Ngan Shing Street, Shatin, New Territories Hong Kong; 2 Department of Anatomical and Cellular Pathology, The Chinese University of Hong Kong, Prince of Wales Hospital, 30-32 Ngan Shing Street, Shatin, New Territories Hong Kong

**Keywords:** Mediastinum, Rhabdomyosarcoma, Mediastinal tumor, Pediatric, Sternotomy

## Abstract

**Introduction:**

Alveolar rhabdomyosarcomas of the mediastinum in children are rarely reported. Multimodality therapy including chemotherapy, surgery and radiotherapy make up the backbone of the treatment of childhood rhabdomyosarcomas. Complete resection whenever achievable is an important prognostic factor. However, complete resection of tumors in the mediastinum often poses a unique challenge to thoracic surgeons due to their close proximity to important neurovascular structures. Complete resection may not always be possible and judicious peri-operative planning and preparation are required to avoid creating unnecessary surgical morbidities resulting in delay of adjuvant therapy.

**Case presentation:**

A 4-month-old Chinese baby boy was presented to our hospital with stridor, shortness of breath and episodes of cyanosis. Imaging studies found an anterior mediastinal mass compressing the trachea and other neurovascular structures and he was diagnosed to have alveolar rhabdomyosarcoma. Our patient received upfront chemotherapy and subsequently open resection of the mass was attempted via median sternotomy. Intraoperatively, the mass had invaded into the great vessels, precluding a complete resection. Debulking surgery was performed instead and our patient received timely postoperative chemoradiotherapy.

**Conclusions:**

We report a rare case of childhood alveolar rhabdomyosarcoma of the mediastinum with vascular invasion treated with chemoradiotherapy and debulking surgery. Complete resection was not possible due to the close proximity to the great vessels. Different surgical approaches to the mediastinum have been reported in adults and children alike. Regardless of the surgical access, the treatment of childhood rhabdomyosarcomas should be individualized, with careful balance between surgical clearance and surgical morbidity.

## Introduction

Rhabdomyosarcoma (RMS) is the commonest soft tissue sarcoma in children and adolescents. Around 20% of RMS is alveolar type, and is associated with poorer prognosis. Treatment of RMS varies widely according to histology, site and age of the patient. Despite the advent of combined chemotherapy, complete surgical resection remains an important prognostic factor in the overall management of RMS. However, tumors in the mediastinum present a unique challenge to surgeons due to their intimate relationship with various important neurovascular structures. RMS in the mediastinum in pediatric patients is rarely reported and experiences with resection of cervicothoracic region tumors in childhood in general have been limited. In this report, we present a rare case of alveolar RMS of the anterior mediastinum with vascular invasion in a baby, and discuss the various considerations in the surgical approach for children with mediastinal tumors.

## Case presentation

**Fig. 1:**
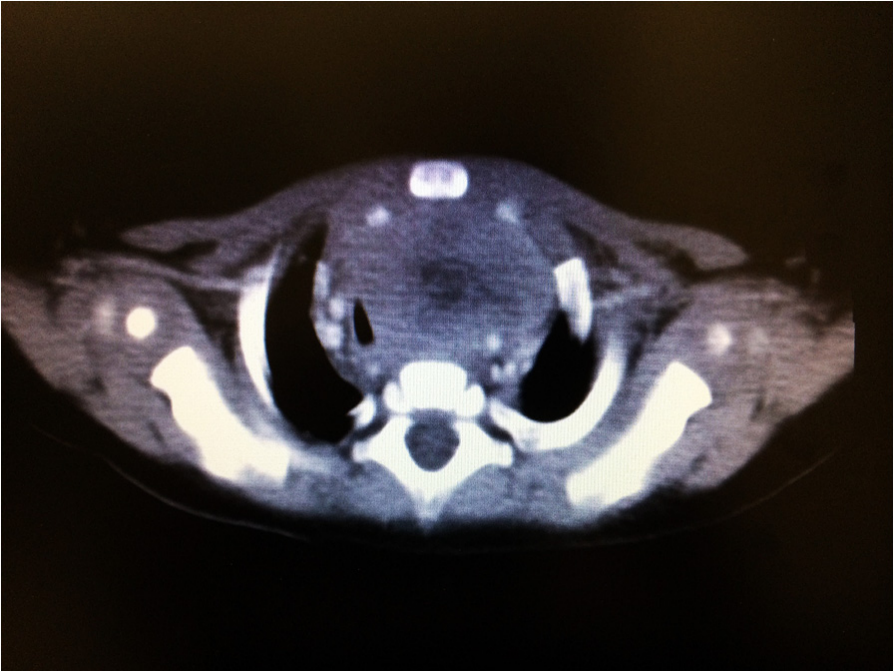
Computed tomographic view of the anterior mediastinal mass compressing and displacing the trachea and great vessels

**Fig. 2:**
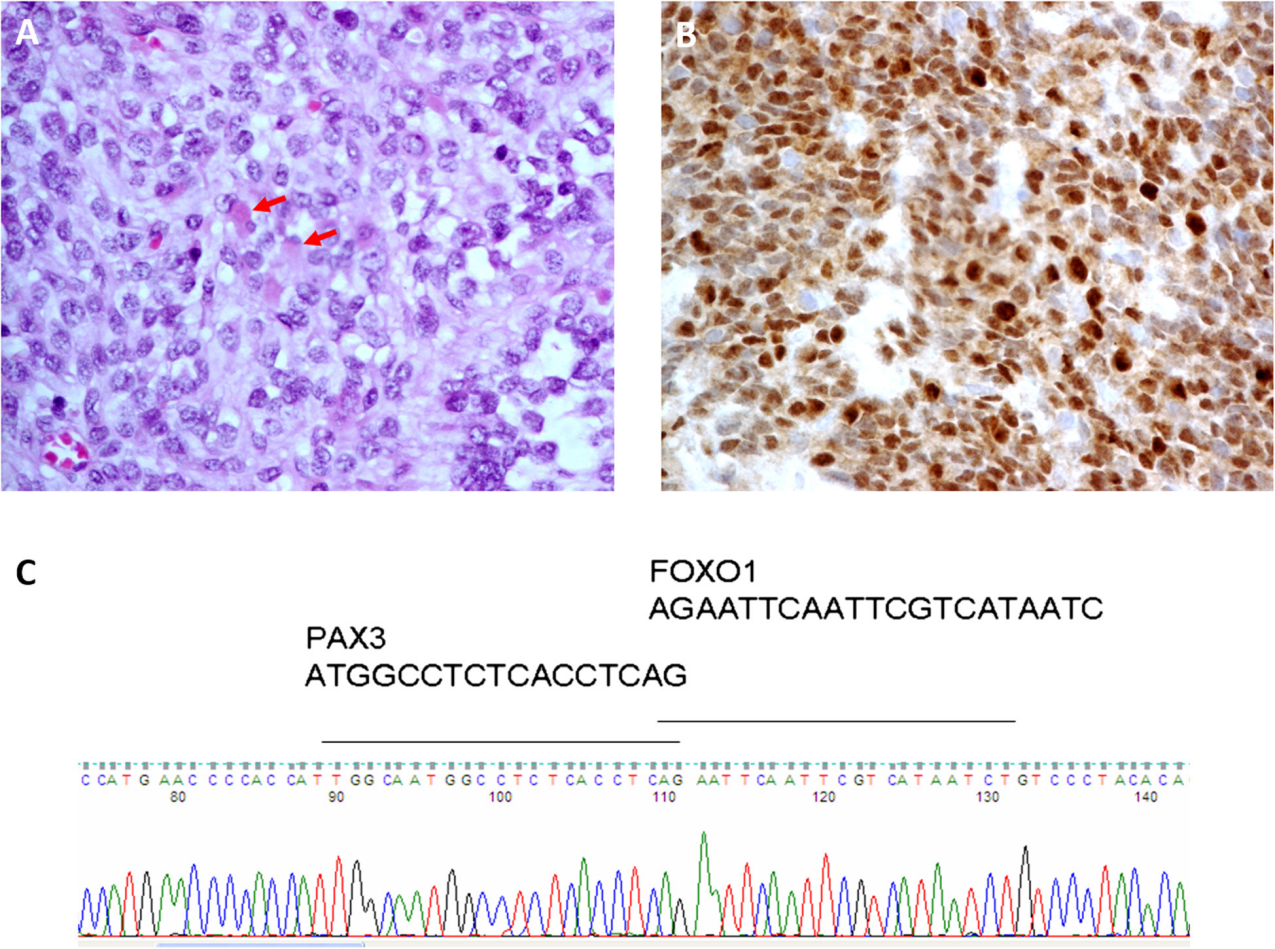
**a** The small round cell tumor arranged mainly in a predominantly solid pattern with a vague alveolar pattern noted focally (in the *lower right* portion of the image). The majority of the tumor cells are primitive in appearance. Occasional tumor cells with more abundant brightly fibrillar eosinophilic cytoplasm, consistent with rhabdomyoblasts (as indicated by *arrows*) are noted. (Hematoxylin and eosin stain, original magnification ×400). **b** On immunohistochemistry, the tumor cells are positive for myogenin and indicative of rhabdomyogenic differentiation. (Immunohistochemical staining for myogenin, original magnification ×400). **c** Typical fusion transcript of PAX3-FOXO1 was detected by reverse transcription polymerase chain reaction, confirming the diagnosis of alveolar rhabdomyosarcoma

A 4-month-old Chinese baby boy had experienced noisy breathing for 2 months. He presented to the pediatrics unit with increasing shortness of breath with episodes of cyanosis. A computed tomography (CT) scan of his chest with contrast enhancement revealed a 7 × 3 × 5cm anterior mediastinal mass extending to the cervical region, compressing the trachea and veins as well as displacing the aortic arch posteriorly (Fig. 1). An incisional biopsy of the mass via an incision over the left cervical strap muscles showed infiltrative loose clusters of tumor cells among fibrous stroma. The tumor cells showed moderate pleomorphism, inconspicious nucleoli and scanty indistinct cytoplasm. No rhabdomyoblasts were seen and no alveolar pattern or teratomatous component was identified. The undifferentiated morphology was more compatible with alveolar rhabdomyosarcoma. The cells stained strongly positive for vimentin, desmin and myogenin. Total RNA was extracted from the tissue and reverse transcription polymerase chain reaction (RT-PCR) detected PAX3/FOXO1 fusion transcript, confirming the diagnosis of alveolar-type rhabdomyosarcoma (Fig. 2).

Ultrasonography of the abdomen, a bone scan and bone marrow study did not reveal any metastatic lesions. Vincristine, actinomycin and cyclophosphamide were commenced, with radiological evidence of tumor regression. After 11 weeks of chemotherapy, a CT scan of his chest showed good response to chemotherapy with shrinkage of the tumor. Our patient subsequently underwent an exploratory median sternotomy at the age of 8 months.

**Fig. 3:**
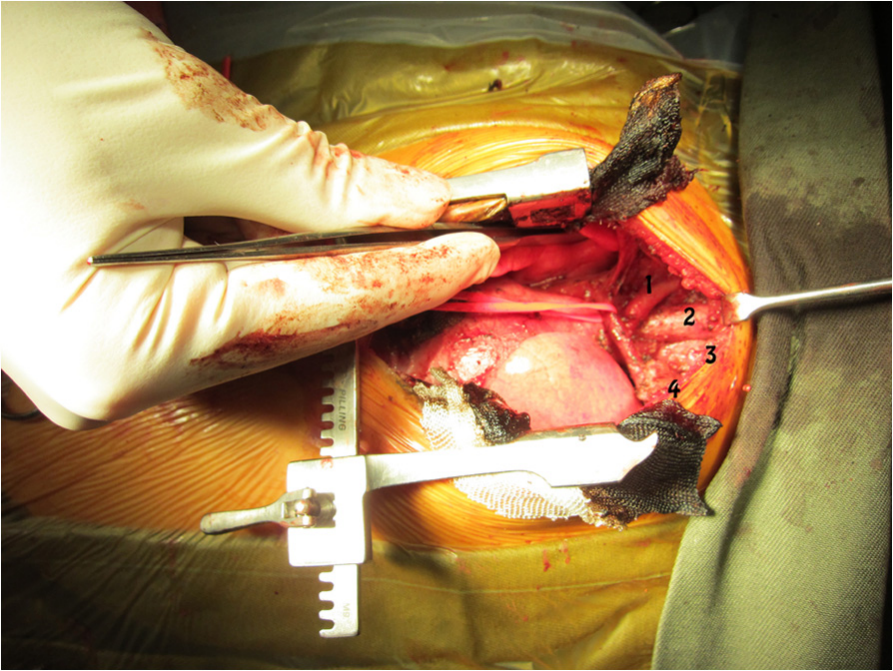
Intraoperative view of the mediastinal bed following resection showing the slung brachiocephalic vein, brachiocephalic artery (labeled *1*), trachea (*2*), esophagus (*3*), and tumor encasing the left brachiocephalic vein and left common carotid artery (*4*)

A hard mass was found at the left superior part of the anterior mediastinum along with a sizeable thymus. The mass spanned from the lower border of the brachiocephalic vein to the neck and from the midline to the area between the left brachiocephalic vein and left common carotid artery. Total thymectomy with preservation of the bilateral phrenic nerves was performed. Complete excision of the tumor was attempted, but failed due to tumor invasion of the left brachiocephalic vein and the left common carotid artery. Debulking of the tumor was performed leaving behind a small volume of residual tumor in the area adjacent to the invaded great vessels (Fig. 3). The site of the involved margin was marked with clips.

**Fig. 4:**
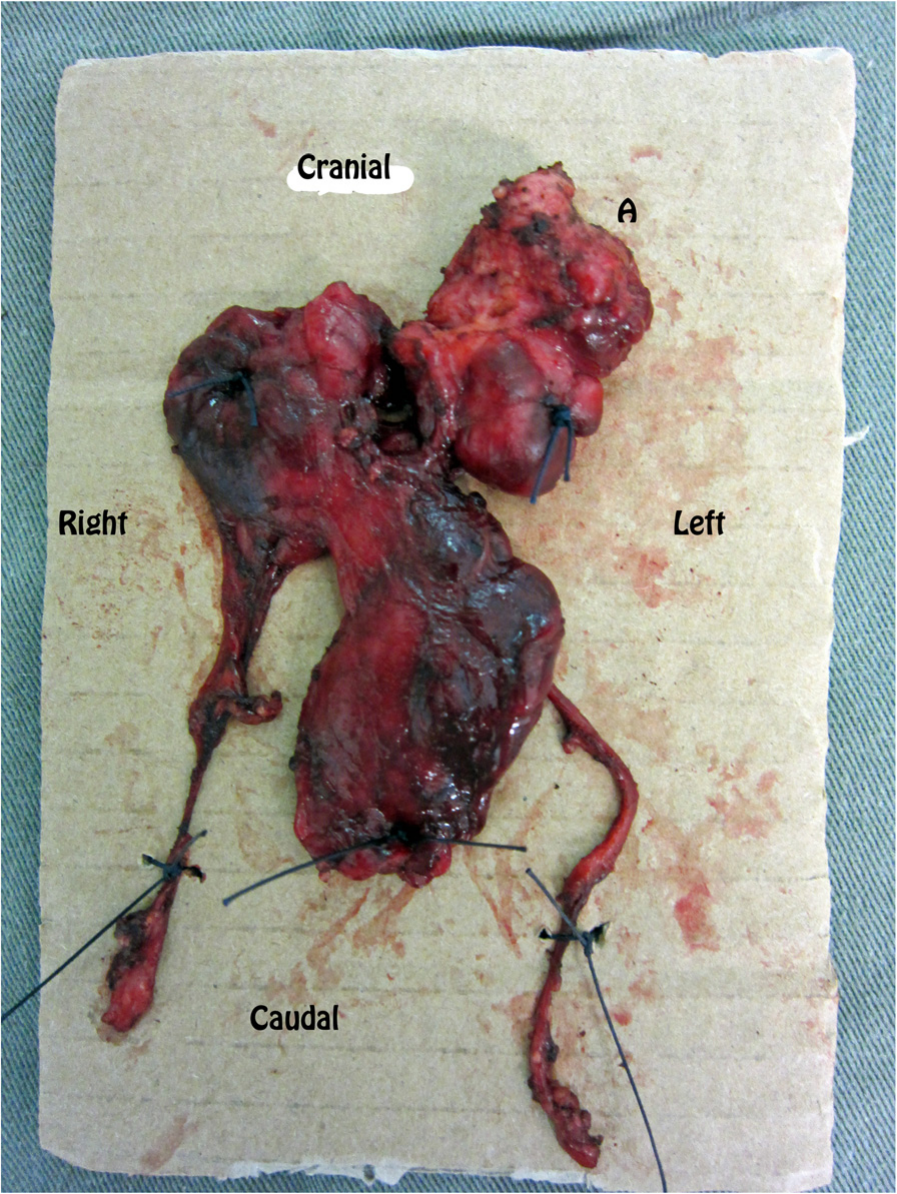
Resected specimen containing the bulky thymus and the mediastinal tumor. The tumor is labeled *A*, with the orientation of the mass marked

A pathologic examination revealed an 8 × 5.5 × 0.8cm tumor invading the thymus, with associated thyroid tissue and skeletal muscle fibers (Fig. 4). Microscopic examination was consistent with rhabdomyosarcoma.

Radiotherapy with a dose of 50.4 Gy was given to the marked area of residual tumor. Unfortunately, a subsequent CT scan 9 months postoperatively and at week 39 of chemotherapy revealed tumor recurrence around the surgical clip site, confirmed by biopsy. The tumor progressed rapidly despite further therapy and our patient ultimately developed respiratory failure and died at the age of 3.

## Discussion

Rhabdomyosarcoma (RMS) is the commonest soft tissue sarcoma in children [1]. The commonest histological variants of RMS are the embryonal (ERMS) and alveolar (ARMS) subtypes, the latter being less common and associated with poorer prognosis. RMS is comparatively rare in adults, only accounting for <1% of all adult solid tumor malignancies while about half of pediatric soft tissue sarcomas are RMS, with slight male predominance. More than two thirds of pediatric RMS is diagnosed before the age of 6. Common sites of occurrence for RMS include the head and neck region, followed by the genitourinary system and the extremities. Mediastinal rhabdomyosarcomas are rare, and are often related to germ cell or teratomatous tumors. To our knowledge only a handful of mediastinal alveolar rhabdomyosarcomas have been reported. Mediastinal rhabdomyosarcomas tend to be larger and more locally invasive than RMS in other regions and are associated with poorer prognosis, with the majority of the cases reported to have early local and distant recurrence post resection.

Specific chromosomal translocations are detected in around 80% of cases of ARMS. Translocation t(2;13)(q35;q14) occurs in 60% of ARMS, and translocation t(1;13)(p36;q14) occurs in approximately 20%. These translocations result in the expression of transcription factors PAX3-FOXO1 or PAX7-FOXO1, which induce angiogenesis and activate cell proliferation. The prognostic value of the presence of fusion gene PAX3-FOXO1 in alveolar rhabdomyosarcoma is still under debate and a latest meta-analysis did not find significant difference in overall survival between patients with the positive and negative fusion gene. Indeed, better quality studies are needed to validate its prognostic significance [2].

The treatment of RMS has evolved over decades. Multimodality treatment protocols devised by large international cooperative groups such as the Intergroup Rhabdomyosarcoma Study Group (IRSG) have improved cure rates for RMS in children from 25% in the 1970s to 70% in the 1990s [3, 4]. This is mainly attributed to improved combined chemotherapy, better and earlier staging and diagnosis, as well as improving local treatment options including radiotherapy and surgery. The chemotherapy backbone of RMS therapy has been vincristine, actinomycin and cyclophosphamide. Complete resection of the tumor should be considered for all localized disease. While diagnostic biopsy combined with neoadjuvant therapy before definitive local treatment is an alternative for initially unresectable disease, the role of debulking and second-look surgery still remain uncertain.

Nonetheless, complete resection of tumors in the cervicothoracic region remains a challenge to thoracic surgeons due to their close relationships with important neurovascular and lymphoid structures. In order to balance completeness of resection with debilitating postoperative morbidity, comprehensive preoperative planning, adequate surgical exposure and meticulous dissection are often required for favorable outcomes. To date, experiences with resection of childhood mediastinal tumors have been limited to case series and reports [1, 3, 5].

Magnetic resonance imaging (MRI) is recognized to be superior to CT in the assessment of tumor relations with adjacent soft tissues. Various adult case series and reports of mediastinal sarcomas had MRI performed to assess for degree of tumor encasement with respect to adjacent great vessels. Vascular involvement is considered absent if there is a less than 180 degree circumferential relationship or a clear plane can be seen between the tumor and vessel [6]. Nonetheless, preoperative radiology may not always identify important vascular invasion. In a case series published recently by Christison-Lagay *et al*., a case of tumor invasion into pulmonary artery was not identified until open surgery, ultimately resulting in aborted surgery [5]. In our case, preoperative CT also did not show conclusive evidence of tumor invasion into the great vessels, and in view of the considerable tumor shrinkage post neoadjuvant chemotherapy, resection surgery was hence performed for our patient. No MRI was performed for this baby, as the risk of general anesthesia, with a mediastinal mass compressing the trachea, would have outweighed the benefit of the scan. Worthy of note, the need for sedation or general anesthesia in young children for MRI, in our view, unlike RMS in other regions, carries a high risk of respiratory failure in mediastinal RMS with visible tracheal compression.

Methods of surgical approach and preservation of important neurovascular structures have been discussed and published extensively in studies relating to resection of adult cervicothoracic tumors. In contrast, similar reports are very limited for childhood mediastinal tumors. Sauvat *et al*. performed the transmanubrial osteomuscular-sparing technique popularized by Grunenwald to achieve more than 90% gross total resections in four children with cervicothoracic neuroblastomas [7, 8]. This approach is considered an alternative to Dartevelle’s anterior transcervical-thoracic approach with reported lower incidence of shoulder girdle dysfunction due to preservation of the clavicle and sternoclavicular joint in adult lung cancer series [9, 10]. To date, the largest series concerning childhood mediastinal tumors was published in 2014 by Christison-Lagay *et al*. from the Sloan-Kettering Cancer Center in New York [5]. They performed “trap-door” and “clam shell” approaches to resection of various extensive cervicothoracic tumors, mostly neuroblastomas, in 17 children with age ranging from 9 month to 29.6 years. Total or near gross total resection was achieved in 94% of patients. One case was unresectable due to invasion to the pulmonary artery. No great vessel reconstruction was performed in their experience. Two patients had patch angioplasty done for the brachiocephalic vein. Outcomes were reported to be good with no chylothorax or vascular injury, and absence of long-term neurological sequel. In our case, we were able to clearly delineate, via a median sternotomy, that the tumor was adherent to the left common carotid artery and closely related to the subclavian artery. So far, there has been no study reporting vascular resection and reconstruction of great vessels in children <1 year old with sarcomas. Isolated reports of left subclavian artery transfers and resection of Kommerell’s diverticulum can be found in the literature, but childhood great vessels reconstruction post tumor resection remains largely unexplored with very little known on the topic [11]. We decided not to attempt gross resection with multiple great vessel resections in our 8-month-old child, taking into account the considerable risks and lack of supporting literature. The need to extend the median sternotomy or convert to a trap-door-type incision was therefore not necessary as this could have potentially introduced further morbidities, which may have delayed further chemoradiotherapy.

## Conclusions

In conclusion, the management of mediastinal sarcoma remains challenging to both oncologists and surgeons. Reports of surgical resection in the literature for childhood, in particular childhood mediastinal rhabdomyosarcoma, are rare. Although complete resection is desirable, a proportion achieves only near-complete resection. Whatever the surgical access, some infant mediastinal tumors are found unresectable intraoperatively, usually because of vascular invasion. The risk and benefit between achieving complete resection and postoperative morbidity leading to delays in chemoradiotherapy must be weighed and balanced accordingly. Ultimately, the management of each case of mediastinal sarcoma should be individualized and multidisciplinary.

## Consent

Written informed consent was obtained from the patient’s legal guardian(s) for publication of this case report and any accompanying images. A copy of the written consent is available for review by the Editor-in-Chief of this journal.

## Abbreviations

ARMS: alveolar rhabdomyosarcoma
CT: computed tomography
ERMS: embryonal rhabdomyosarcoma
IRSG: Intergroup Rhabdomyosarcoma Study Group
MRI: magnetic resonance imaging
RMS: rhabdomyosarcoma
RT-PCR: reverse transcription polymerase chain reaction
